# Altered Neural and Behavioral Dynamics in Huntington's Disease: An Entropy Conservation Approach

**DOI:** 10.1371/journal.pone.0030879

**Published:** 2012-01-23

**Authors:** S. Lee Hong, Scott J. Barton, George V. Rebec

**Affiliations:** 1 Department of Kinesiology, Indiana University, Bloomington, Indiana, United States of America; 2 Department of Psychological and Brain Sciences, Indiana University, Bloomington, Indiana, United States of America; University of Maribor, Slovenia

## Abstract

**Background:**

Huntington's disease (HD) is an inherited condition that results in neurodegeneration of the striatum, the forebrain structure that processes cortical information for behavioral output. In the R6/2 transgenic mouse model of HD, striatal neurons exhibit aberrant firing patterns that are coupled with reduced flexibility in the motor system. The aim of this study was to test the patterns of unpredictability in brain and behavior in wild-type (WT) and R6/2 mice.

**Methodology/Principal Findings:**

Striatal local field potentials (LFP) were recorded from 18 WT and 17 R6/2 mice (aged 8–11 weeks) while the mice were exploring a plus-shaped maze. We targeted LFP activity for up to 2 s before and 2 s after each choice-point entry. Approximate Entropy (ApEn) was calculated for LFPs and Shannon Entropy was used to measure the probability of arm choice, as well as the likelihood of making consecutive 90-degree turns in the maze. We found that although the total number of choice-point crossings and entropy of arm-choice probability was similar in both groups, R6/2 mice had more predictable behavioral responses (i.e., were less likely to make 90-degree turns and perform them in alternation with running straight down the same arm), while exhibiting more unpredictable striatal activity, as indicated by higher ApEn values. In both WT and R6/2 mice, however, behavioral unpredictability was negatively correlated with LFP ApEn.

**Conclusions/Significance:**

HD results in a perseverative exploration of the environment, occurring in concert with more unpredictable brain activity. Our results support the entropy conservation hypothesis in which unpredictable behavioral patterns are coupled with more predictable brain activation patterns, suggesting that this may be a fundamental process unaffected by HD.

## Introduction

Huntington's disease (HD) is a fatal inherited condition characterized by severe cognitive, emotional, and motor symptoms. The striatum, a forebrain structure that processes cortical information for behavioral output, is a key HD target that undergoes pronounced neurodegeneration. Long before striatal neurons die, however, they become dysfunctional, as seen in symptomatic R6/2 mice, a widely used transgenic model of HD. R6/2 striatal neurons show altered firing patterns, including a decrease in burst activity and a loss of correlated firing between simultaneously recorded neuron pairs relative to wild-type (WT) mice [1]. Behaviorally, R6/2 mice are less likely than WT to turn right or left in a plus maze, an indication of motor inflexibility [2].

An important question that this study seeks to address is how brain and behavior interactions are altered in R6/2 mice. This is a particularly important issue for developing drugs or other treatment options in HD aimed at reversing or attenuating abnormal neural firing patterns. Here, we examined patterns of brain activation in behaving R6/2 and WT mice, with emphasis on the dynamics of local field potentials (LFPs) in the striatum before, during, and after the animal chooses to enter an arm within the plus maze. We also quantified the pattern of arm-choice selections.

Measures that quantify the probability of different events can provide a common metric for the quantification of patterns of both brain and behavior, using the framework of entropy and uncertainty. Such an approach detects the likelihood of the occurrence of specific behavioral events and the probability that sequences recur within brain activation signals over a given period of time. This allows us to examine the hypothesis of entropy conservation in brain-behavior relationships [3]–[6]. The idea of entropy conservation is essentially a single resource model across the brain, whereby only a limited number of configurations are possible [3], [6]. As a result, as these “degrees of freedom” within the brain are being diverted to perform a task, the remaining resources to engage in other functions are reduced. The inherent bridge between degrees of freedom, probability, and uncertainty allow entropy to be used as a measure of the amount of information required to describe the behavior of the system, while also providing an indirect measure of the number of different configurations taken on by the system over a given period of time.

Effectively, our goal was to ask the following questions: (1) *are the behaviors of R6/2 mice less unpredictable than the WT*; (2) *will the brain activation patterns in R6/2 mice be more unpredictable than those in WT*; and (3) *does the unpredictability of behavior increase when brain activation patterns are more predictable (and vice versa)*? We also assess whether the brain-behavior unpredictability issue holds across both types of mice or just one type. Brain activity was assessed using dynamical patterns of striatal local field potentials (LFPs), the collective activity of large neuronal populations, measured with emphasis on the choices made by the mice as they navigated a plus maze.

## Results

We collected LFPs from 18 WT and 17 R6/2 mice as they navigated the plus maze for periods of 30 min (data from one R6/2 mouse was not analyzed as the mouse did not have any choice point crossings throughout the 30-min trial). Patterns of behavior were obtained by tracking the movements of the mouse within the maze and marking when the mouse crossed the choice point lines in the center (both entry into and exit out of a given arm).

### Behavioral Measures

**Figure 1:**
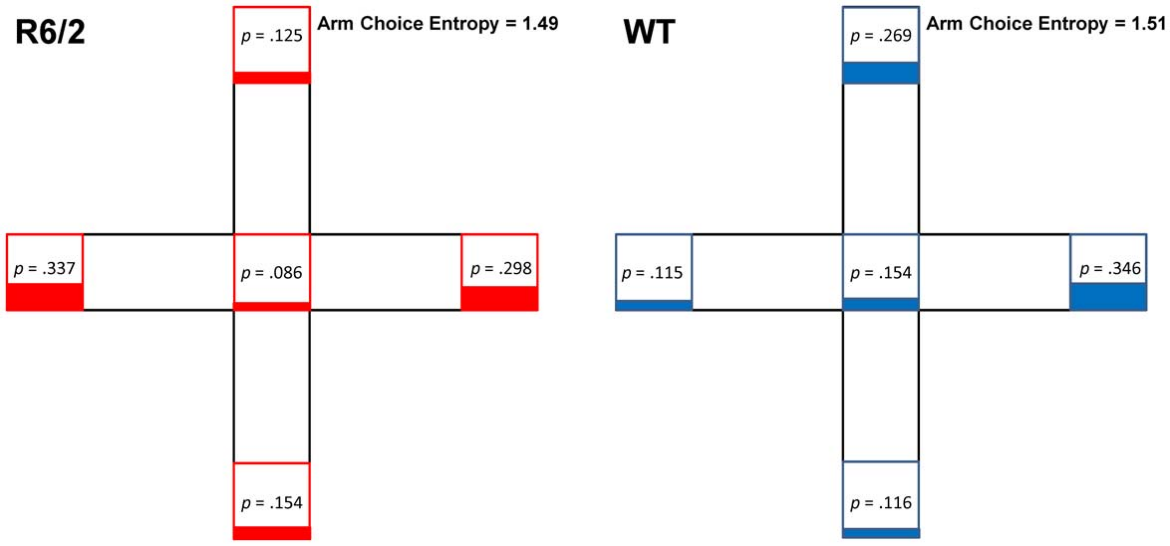
Exemplar probability distribution of arm choices. Data presented are obtained from a single trial, with one animal from each group. The arm choice entropy values (calculated using Equation 1) are also provided.

Arm choice entropy was slightly, but not significantly higher in WT in comparison to R6/2 mice. Exemplar probability distributions in the plus maze from a single mouse from each group are presented in Figure 1. R6/2 mice were nearly twice as likely to run straight along the same arm, while the WT mice were more likely to make 90-degree turns and alternate locomotor behaviors on consecutive choices, as evidenced by the higher lag-1 autocorrelation values. The number of choice point crossings and arm choice entropy were not significantly different between groups. Results from the behavioral measures are summarized in Table 1.

**Table 1:** Summary of statistical results for the behavioral measures.

Variable	WT (*M*±*SE*)	HD (*M*±*SE*)	*F* (1,33)	*p*	η_p_^2^
Number of Choice Point Crossings	112±12	90±12	1.84	.184	.053
Arm Choice Entropy	1.53±0.02	1.47±0.03	2.55	.120	.072
Probability of Remaining in the Same Arm	0.47±0.04	0.74±0.04	19.44	<.001	.371
Lag-1 Autocorrelation	0.51±0.05	0.33±0.05	7.43	.010	.184

### Brain Activity Patterns

**Figure 2:**
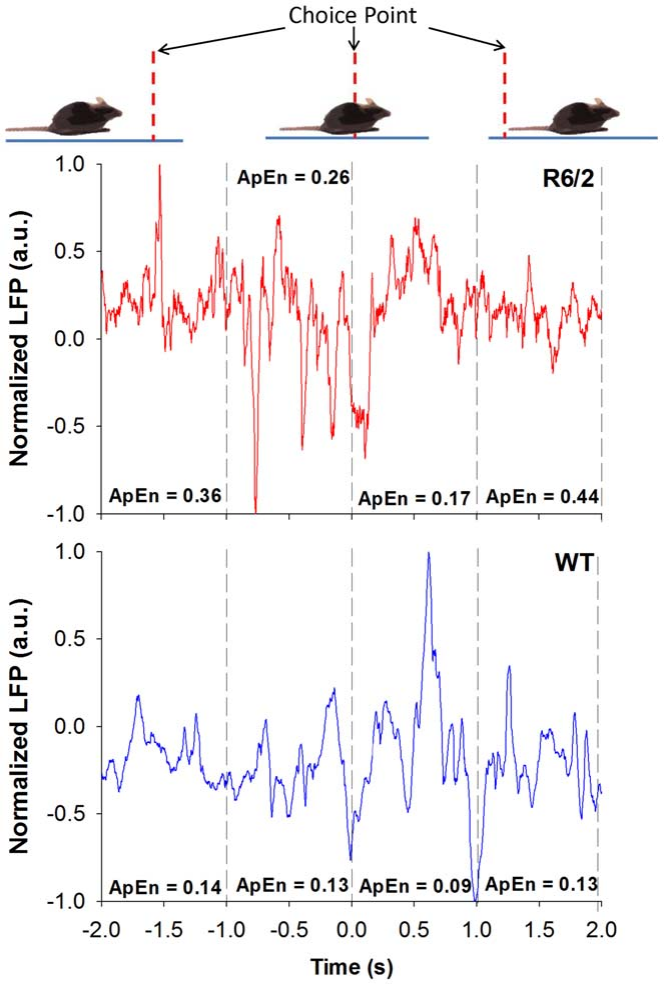
Exemplar LFP dynamics around the choice point. These are data obtained from a single, choice-point crossing during a trial for a single animal within each group. Both choices represent the animal crossing the choice point and remaining in the same arm. The ApEn values within the time windows of A) 2 s to 1 s prior; B) 1 s to 0 s prior; C) 0 s to 1 s after; and D) 1 s to 2 s after crossing the choice point are also illustrated. Note that the data have been normalized to fall between −1 and 1, so as to highlight the differences in dynamics (hence, the scale on the *y*-axis is in arbitrary units, a.u.).

Micro-electrodes were chronically implanted in striatum in order to obtain local field potential (LFP) signals, which were extracted at critical points surrounding each choice-point crossing: A) 2 s to 1 s prior; B) 1 s to 0 s prior; C) 0 s to 1 s after; and D) 1 s to 2 s after crossing the choice point. We analyzed the patterns of LFP activity by dividing them into two different components: 1) amplitude, measured using RMS; and 2) dynamics, measured using Approximate Entropy (ApEn). Exemplar LFP dynamics obtained from a single mouse during a choice-point crossing is presented in Figure 2.

### LFP Signal Amplitude

**Figure 3:**
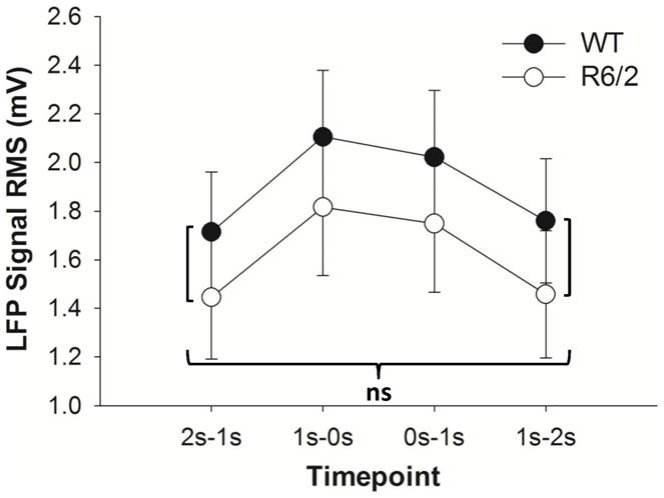
LFP signal amplitude around the choice points. Only a significant time effect was observed. Post-hoc analysis of the main effect of time revealed that all of the Bonferroni-corrected pairwise comparisons for the RMS values at the different time points were significant, with the exception of the comparison between 2-1s prior and 1-2s after the choice point, marked **ns** for not significant. Data are averaged across all choice point crossings and animals within each group; error bars reflect one standard error of the mean.

A significant effect of Time (*F*(1.5,49.6) = 74.92; *p*<0.001; η_p_^2^ = .694) was found for the RMS values of the LFPs. Bonferroni corrected post-hoc examination of the main effect of Time revealed that the differences for all of the pairwise comparisons were significant (*p*<0.01), with the exception of the comparison between 2 s–1 s prior and 1 s–2 s after the choice. RMS values were observed to increase up until the choice point, and then decline (see Figure 3). The Time×Group interaction (*F*(1.5,49.6) = 0.12; *p* = .828; η_p_^2^ = .004) and Group effect (*F*(1,33) = 0.57; *p* = .455; η_p_^2^ = .017) were not significant.

### LFP Dynamics

**Figure 4:**
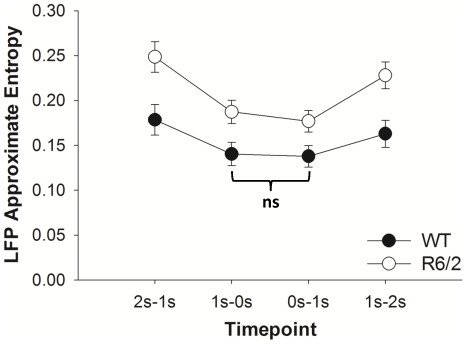
ApEn values for the LFP dynamics around the choice points, illustrating the significant Time×Group interaction. All the Bonferroni-corrected pairwise comparisons were significant, with the exception of the comparison marked **ns** for not significant. Both the main effects of Group and Time were significant. Data are averaged across all choice-point crossings and animals within each group; error bars reflect one standard error of the mean.

A significant Time×Group interaction was observed (*F*(1.6,53.7) = 7.61; *p* = .009; η_p_^2^ = .187) for the ApEn of the LFP signal. Bonferroni corrected post-hoc analyses of the interaction revealed significant differences across all pairwise comparisons (*p*<.01), with the exception of the ApEn during 1-0 s prior and 0-1 s after the choice point crossing for WT mice. The interaction is illustrated in Figure 4. We also observed a significant Group effect (*F*(1,33) = 7.68; *p* = 0.002; η_p_^2^ = .189) on ApEn values of the LFPs, where there was significantly more unpredictability in the dynamics of R6/2 (*M*±*SE* = 0.21±0.014) compared to WT mice (*M*±*SE* = 0.16±0.014).

A significant effect of Time on the ApEn of the LFP signal (*F*(1.6,53.7) = 99.13; *p*<.0001; η_p_^2^ = .750) was found, and post-hoc tests revealed significant differences across all pairwise comparisons (*p*<.01). Here, the LFP ApEn values were highest during the period from 2-1 s prior to the choice-point crossing. These values continued to decline up until 1 s after the choice-point crossing, and then increased in the ensuing 1–2 s.

### Brain-Behavior Relationships

**Figure 5:**
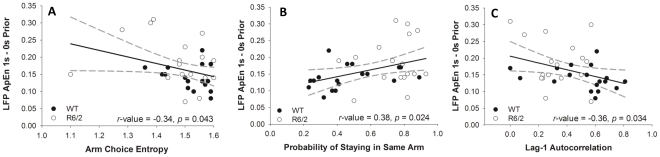
Significant relationships between the unpredictability in brain and behavior. The following panels represent correlations between striatal LFP ApEn values and: A) Arm Choice Entropy; B) Probability of Remaining within the Same Arm; and C) Lag-1 Autocorrelation. The solid black line represents the line of best fit while dashed gray lines reflect the 95% confidence interval.

Partial and zero-order correlations were used to test for significant relationships between brain (LFP ApEn and RMS 1-0s prior) and behavioral variables (Arm Choice Entropy, Probability of Remaining in the Same Arm, and Lag-1 Autocorrelation of Arm Choice). All the partial correlations controlling for mouse type were not significant (*p*>0.05; largest *r-value* = −.276; smallest *p* = 0.114, between ApEn and the number of choice point crossings). All the zero order correlations between ApEn and the behavioral variables were significant (see Figure 5), but, not for RMS (*p*>0.05; largest *r-value* = −.232; smallest *p* = 0.179, between RMS and probability of running straight and remaining in the same arm).

## Discussion

Behaviorally, we found two key differences between the HD and WT groups. First, the sequence of choice point crossings was significantly different between groups (answering “yes” to question 1). This was evident in the lower likelihood that a WT mouse would remain within the same arm and by the higher lag-1 autocorrelation of choices in the WT. Both of these findings are consistent with the existing findings that HD mice are less likely to make 90 degree turns within a plus maze. In addition to the existing research, our data show that WT mice are more likely to alternate between running straight and making 90-degree turns. These findings support the hypothesis that HD results in decreased flexibility in the motor system [2].

Our LFP data revealed a universal difference between the two groups. We found greater unpredictability in striatal LFP activity in the HD mice (answering “yes” to question 2), a difference that was maintained across all time points around the choice events. One explanation for this finding is that the loss of correlated firing between neurons in R6/2 mice [1] would lead to greater independence across the neuronal population that generates the LFPs. This reduced correlation or “synchrony” in the firing of individual neurons would unfold in time as a more unpredictable sequence at the population level, hence, the higher ApEn values indicating greater unpredictability in the signal.

A common scaling of brain-behavior relationships was observed, as seen in the significant correlations between the unpredictability of LFP dynamics and behavior (answering “yes” to question 3). Across all the dependent variables, LFP dynamics were seen to be more unpredictable when behavior was more predictable. This is evidenced by the increased ApEn values of the striatal LFPs when Arm Choice entropy was lower. Similarly, ApEn values were lower when: A) the animal had a high likelihood of remaining in the same arm (and running straight); and B) subsequent 90 degree turns were less likely. These findings are consistent with the entropy conservation hypothesis [3]–[6], showing that a conservation of entropy can be observed as a complementary interaction across brain and behavior [7].

An explanation for this relationship is the idea of a limited level of entropy across the entire brain, as presented in Smotherman et al. [6]. Using cocaine-fed rat pups, these authors found that a decreased behavioral response to environmental stimuli could be explained by the redistribution of entropy across the brain, while the total entropy of activity within the brain remained constant. Our findings support this single-resource viewpoint of the brain, albeit inferred, based on the patterns of brain and behavior. Our results suggest, however, that behavioral patterns could serve as a proxy measure for altered input-output relationships [8] that are reflective of changes occurring across the entire brain with constant entropy [5], [6].

Interestingly, significant brain-behavior relationships were only observed in the zero-order correlations, suggesting that similarities in brain and behavior that were common across both types of mice. This is perhaps an indication that the relationship between the unpredictability of brain and behavior is an aspect of the neurobehavioral system not significantly impacted by HD. First, from the perspective of distributions of behavior, both groups explored the maze similarly as indicated by the lack of statistically significant differences in the number of choice-point crossings and the entropy of arm-choice selections. Interestingly, behavioral differences between HD and WT mice only become apparent when the sequential properties of maze exploration are examined. Second, the strength of the LFP signal from the striatum did not differ and both types of mice exhibited increases in signal amplitude whenever faced with a choice point. Much like the behavioral measures, the higher amplitude-independent unpredictability within the LFP sequence is what distinguishes HD from WT mice.

Overall, the characteristic feature of plus-maze exploration in R6/2 mice is a perseverative pattern of locomotion accompanied by unpredictable dynamics in striatal LFPs. However, striatal LFP dynamics do not directly reflect moment-to-moment choice decisions, as we did not observe direct one-to-one brain-behavior relationships at every choice-point crossing. Instead, the relationship between unpredictability across brain and behavior only emerges after a distribution of behaviors has been obtained. This phenomenon parallels the work of Schultz [9] and Fiorillo et al. [10], who found neural correlates of brain activity in response to prediction of reward after a probability of reward has been defined over numerous repeated trials.

Clinically, the finding that both WT and HD mice share a similar brain-behavior relationship raises the possibility of attenuating symptoms through new interventions. One possibility is to alter brain activity dynamics pharmacologically, for example by adjusting dopamine transmission through the administration of L-DOPA, an approach that has short-term benefits, but deleterious long-term effects [11]. A second possibility would be to employ deep-brain stimulation methods that have been found to be effective in the treatment of Parkinson's disease symptoms [12]. Across both approaches, the goal would be to reduce the unpredictability or “noise” within striatal circuit activity.

### Conclusions

This study tested the effects of HD on patterns of brain and behavior, answering the following research questions:

1. Are the behaviors of R6/2 mice less unpredictable than WT? *Yes*. These HD mice are less likely to make 90 degree turns in the plus maze and are also less likely to make consecutive turns in the exploration sequence. But, the number of choice point crossings and the entropy of the distribution of arm choices were not different between the groups. *This suggests that HD mice exhibit perseverative exploration, an effect that is revealed in the sequence of choices, rather than the absolute count of exploration events.*
2. Will the brain activation patterns in R6/2 mice be more unpredictable that the WT? *Yes*. Striatal LFP activity patterns were more unpredictable across all time points around the choice event. But the pattern of change in LFP dynamics in response to the choice were relatively similar with the exception of the lack of change in the one second immediately prior to and after the choice-point crossing. *This supports the idea that HD mice have less correlated neuronal firing that leads to more unpredictable, noisier brain activity.*
3. Does the unpredictability of behavior increase when brain activation patterns are more predictable (and vice versa)? Does the pattern hold across both types of mice or just one type? *Yes*. Significant correlations were found between brain-activity entropy and arm-choice entropy, the probability of the mouse remaining in the same arm, and lag-1 autocorrelation of arm transitions. These correlations were not significant when separated by group, but when both groups were entered into the analysis, the correlations were significant. *This finding supports the hypothesis of entropy conservation across brain and behavior, suggesting also that this is a fundamental relationship unaffected by HD.*

## Materials and Methods

The R6/2 mouse model of HD expresses exon 1 of the huntingtin gene with an expanded region of ∼140 CAG (glutamine) repeats. This model exhibits behavioral signs of HD as early as 6 to 7 weeks of age [13]. Seventeen R6/2 mice (age 8 to 14 weeks, mean age 10.5±1.9 wks) and 18 wildtype (WT) littermate controls (age 8 to 13 weeks, mean age 10.0±1.9 wks) underwent micro-electrode implantation surgery to measure striatal local field potentials (LFPs).

Mice were anesthetized with an intraperitoneal injection of chloropent (0.4 mL/100 g) and placed in a stereotaxic frame. An incision exposed the skull, and a 1.0 mm diameter hole was drilled unilaterally over striatum (0.5 mm anterior and ±1.5 mm lateral to bregma). The electrode assembly consisted of 8 microwires (Formvar-coated, 25 µm diameter stainless steel) for recording and 2 non-insulated, 50 µm diameter stainless steel ground wires. Each wire was friction-fitted with a gold-plated pin into a hole in a polyphenylene sulfide insulator (Omnetics Connector Corporation, Minneapolis, MN). The 3.0 mm long bundle was slowly lowered (2.5 mm ventral to brain surface) into striatum and permanently attached to the skull using dental acrylic as described with greater detail in Miller et al. [1].

**Figure 6:**
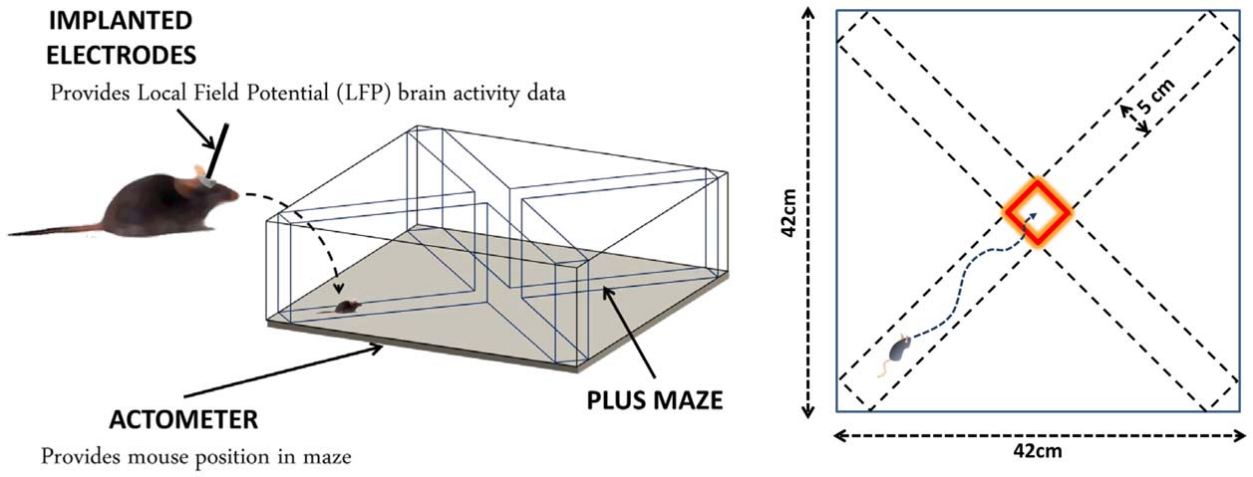
Schematic illustration of experimental setup. Left panel shows the positioning of the mouse in the maze on top of the actometer. Dimensions of the maze are provided in the right panel.

After 10 days of recovery, the electrode assembly was connected to a lightweight flexible wire harness equipped with field-effect transistors that provided unity gain current amplification for each wire, and the animal was placed in an enclosed, plus-shaped maze of transparent Plexiglass (see Figure 6). The maze (arms 25 cm long×5 cm wide with side 30 cm high) was suspended 2 mm over a force-plate actometer [14] that monitored the position of the mouse as it freely explored the maze for 30 min. LFPs, routed through preamplifiers with 1000× gain and 0.7–170 Hz filters, were sampled at 1000 Hz and acquired by a multichannel acquisition processor (Plexon, Dallas, TX, USA). All aspects of animal use were in strict accordance with the National Institutes of Health Guide for the Care and Use of Laboratory Animals and were approved by the local Institutional Animal Care and Use Committee. All efforts were made to minimize suffering and all surgeries were conducted under chloropent anesthesia.

### Data Analysis

The actometer indicated whenever the mouse crossed over the choice point in the maze, whether into or out of the center by placing a timestamp on the LFP data. We isolated crossing conditions in which the mouse had remained in the previous arm for at least 2 s prior to entering the choice point and stayed in the subsequent arm for at least 2 s afterwards. This allowed for the normalization of crossing conditions, while also removing conditions in which the mouse was simply lingering at the choice-point line and creating spurious choice-crossing timestamps. LFP data were then extracted from: A) 2 s to 1 s prior; B) 1 s to 0 s prior; C) 0 s to 1 s after; and D) 1 s to 2 s after crossing the choice point.

#### Dependent Variables - Behavioral Measures

There were 4 behavioral measures obtained: 1) number of choice point crossings; 2) arm choice entropy; 3) probability of staying in the same arm; and 4) lag-1 autocorrelation of same vs. different arm choices. The first behavioral measure was the number of times a given mouse crossed any choice point (both in and out from the center). The second measure, Arm Choice Entropy was calculated based on the probability distribution that a mouse crossed a particular choice point, using the Shannon [15] equation:
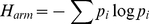
(1)


The third behavioral measure was the probability that the mouse remained in the same arm by running straight after making a choice-point crossing. A shift into a perpendicular arm represented a 90-degree turning movement. The fourth measure of behavior, a lag-1 autocorrelation, was obtained from a time evolution profile of these choice events, where 90-degree changes in running direction were marked differently from events where the mouse ran straight along the same arm. An autocorrelation value of one would mean that the mouse alternated running straight with 90-degree turns from one choice to the next, while an autocorrelation of zero would mean repetitively making 90-degree turns or running straight up-and-down the same arm.

#### Dependent Variables – Brain Measures

Because LFP data were collected from 8 different electrodes, Principal Component Analysis was used to extract a single collective signal. We obtained the data from the 8 electrodes that projected onto the first principal component (i.e., largest eigenvector) that accounted for the most variance in the data set. This approach was used instead of obtaining an ensemble mean across electrodes, as the process of extracting data using Principal Component Analysis simultaneously serves as a noise filter (see Daffertshofer et al. [16]).

The amplitude of the signal within each time window was measured by root mean square (RMS):
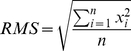
(2)


Unpredictability or irregularity within the dynamics of the unit variance normalized LFP time-series (mean subtracted, divided by standard deviation) was measured by Approximate Entropy (Pincus [17]) summarized as follows:
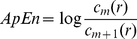
(3)This approach approximates the entropy of a sequence by obtaining the logarithm of the ratio of recurrence counts, *C*, of sequences of lengths *m* and *m*+1, within the data, within the toleration range, *r*. Per the recommendations of Pincus [17], we use the parameters *m* = 2 and *r* = 0.2. If the time-series is predictable, the recurrence count of sequences of length 2 will be close to the recurrence count of sequences of 3 data points, thus, leading to lower ApEn values. An unpredictable time-series will have a much higher rate of recurrence of the shorter sequences in comparison to the longer ones, resulting in higher ApEn values.

### Statistical Analysis

Univariate ANOVA tests for group effects were conducted on the number of choice crossings, arm choice entropy, probability of remaining in the same arm, and lag-1 autocorrelation of arm choices. A mixed-model (4×2) ANOVA was used to measure the event-related LFP signal amplitude and dynamics, with repeated measures on the effect of Time and mouse type as the group effect. Effect sizes were determined using partial eta-squared (η_p_^2^) values. Based on Cohen [18], effects with η_p_^2^ values of .01, .09, and .025 are small, medium, and large effects, respectively. Whenever violations of the sphericity assumption were encountered, a Huynh-Feldt correction for the statistical degrees of freedom was applied. Multiple comparisons were corrected for in post hoc analyses using a Bonferroni correction. To test the relationship between variables, we employed zero-order correlations and partial correlations, controlling for mouse type.

## References

[1] Miller BR, Walker AG, Shah AS, Barton SJ, Rebec GV (2008). Dysregulated information processing by medium spiny neurons in striatum of freely behaving mouse models of Huntington's disease.. Journal of Neurophysiology.

[2] Rebec GV, Barton SJ, Marseilles AM, Collins K (2003). Ascorbate treatment attenuates the Huntington behavioral phenotype in mice.. Neuro Report.

[3] Hong SL (2010). The entropy conservation principle: Applications in human factors and ergonomics.. Nonlinear Dynamics, Psychology, and Life Sciences.

[4] Hong SL, Newell KM (2008). Entropy conservation in the control of human action.. Nonlinear Dynamics, Psychology, and Life Sciences.

[5] Mandell AJ, Selz KA (1997). Entropy conservation as *T_μ_*≈*λ*
^+^
*d_μ_* in neurobiological dynamical systems.. Chaos.

[6] Smotherman WP, Selz KA, Mandell AJ (1996). Dynamical entropy is conserved during cocaine-induced changes in fetal rat motor patterns.. Psychoneuroendocrinology.

[7] Kelso JAS, Engstrøm DA (2006). The complementary nature.

[8] Hong SL, Beck MR (2010). Uncertainty compensation in human attention: Evidence from response times and fixation durations.. PLoS ONE.

[9] Schultz W (1997). Dopamine neurons and their role in reward mechanisms.. Current Opinion in Neurobiology.

[10] Fiorillo CD, Tobler PN, Schultz W (2003). Discrete coding of reward probability and uncertainty by dopamine neurons.. Science.

[11] Hickey MA, Reynolds GP, Morton AJ (2002). The role of dopamine in motor symptoms in the R6/2 transgenic mouse model of Huntington's disease.. Journal of Neurochemistry.

[12] Kern DS, Kumar R (2007). Deep brain stimulation.. The Neurologist.

[13] Mangiarini L, Sathasivam K, Seller M, Cozens B, Harper A (1996). Exon 1 of the HD gene with an expanded CAG repeat is sufficient to cause a progressive neurological phenotype in transgenic mice.. Cell.

[14] Fowler SC, Miller BR, Gaither TW, Johnson MA, Rebec GV (2009). Force-place quantification of progressive behavioral deficits in the R6/2 mouse model of Huntington's disease.. Behavioral Brain Research.

[15] Shannon CE (1948). A mathematical theory of communication.. Bell Syst Tech J.

[16] Daffertshofer A, Lamoth CJC, Meijer OG, Beek PJ (2004). PCA in studying coordination and variability: a tutorial.. Clinical Biomechanics.

[17] Pincus SM (1991). Approximate entropy as a measure of system complexity.. Proceedings of the National Academy of Sciences.

[18] Cohen J (1988). Statistical power analysis for the behavioral sciences (2^nd^ ed.).

